# DNA Methylation Patterns in Cord Blood DNA and Body Size in Childhood

**DOI:** 10.1371/journal.pone.0031821

**Published:** 2012-03-14

**Authors:** Caroline L. Relton, Alexandra Groom, Beate St. Pourcain, Adrian E. Sayers, Daniel C. Swan, Nicholas D. Embleton, Mark S. Pearce, Susan M. Ring, Kate Northstone, Jon H. Tobias, Joseph Trakalo, Andy R. Ness, Seif O. Shaheen, George Davey Smith

**Affiliations:** 1 HNRC, Institute of Genetic Medicine, Newcastle University, Newcastle upon Tyne, United Kingdom; 2 MRC Centre for Causal Analyses in Translational Epidemiology, University of Bristol, Bristol, United Kingdom; 3 Musculoskeletal Research Unit, School of Clinical Sciences, University of Bristol, Bristol, United Kingdom; 4 Bioinformatic Support Unit, Newcastle University, Newcastle upon Tyne, United Kingdom; 5 Newcastle Neonatal Service, Royal Victoria Infirmary, Newcastle upon Tyne, United Kingdom; 6 Institute of Health and Society, Newcastle University, Newcastle upon Tyne, United Kingdom; 7 School of Social and Community Medicine, University of Bristol, Bristol, United Kingdom; 8 Wellcome Trust Centre for Human Genetics, University of Oxford, Oxford, United Kingdom; 9 School of Dental Sciences, University of Bristol, Bristol, United Kingdom; 10 Barts and the London School of Medicine and Dentistry, Queen Mary University of London, London, United Kingdom; Wayne State University, United States of America

## Abstract

**Background:**

Epigenetic markings acquired in early life may have phenotypic consequences later in development through their role in transcriptional regulation with relevance to the developmental origins of diseases including obesity. The goal of this study was to investigate whether DNA methylation levels at birth are associated with body size later in childhood.

**Principal Findings:**

A study design involving two birth cohorts was used to conduct transcription profiling followed by DNA methylation analysis in peripheral blood. Gene expression analysis was undertaken in 24 individuals whose biological samples and clinical data were collected at a mean ± standard deviation (SD) age of 12.35 (0.95) years, the upper and lower tertiles of body mass index (BMI) were compared with a mean (SD) BMI difference of 9.86 (2.37) kg/m^2^. This generated a panel of differentially expressed genes for DNA methylation analysis which was then undertaken in cord blood DNA in 178 individuals with body composition data prospectively collected at a mean (SD) age of 9.83 (0.23) years. Twenty-nine differentially expressed genes (>1.2-fold and *p*<10^−4^) were analysed to determine DNA methylation levels at 1–3 sites per gene. Five genes were unmethylated and DNA methylation in the remaining 24 genes was analysed using linear regression with bootstrapping. Methylation in 9 of the 24 (37.5%) genes studied was associated with at least one index of body composition (BMI, fat mass, lean mass, height) at age 9 years, although only one of these associations remained after correction for multiple testing (*ALPL* with height, *p*_Corrected_ = 0.017).

**Conclusions:**

DNA methylation patterns in cord blood show some association with altered gene expression, body size and composition in childhood. The observed relationship is correlative and despite suggestion of a mechanistic epigenetic link between *in utero* life and later phenotype, further investigation is required to establish causality.

## Introduction

Environmental conditions during development *in utero* and early postnatal life influence health across the lifecourse, including diseases such as obesity, type 2 diabetes and cardiovascular disease [1]–[3]. Epigenetic mechanisms, whereby DNA is modified with downstream influences on gene regulation, may explain how such exposures are recorded with later consequences for growth, development and health through the lifecourse [4]–[7].

Epigenetic patterns, in particular DNA methylation, have been studied in both animal models and human populations with respect to a variety of environmental and lifestyle exposures. Previous work has demonstrated associations between early life exposures and subsequent epigenetic patterns in offspring; between maternal postnatal behaviours in rats and DNA methylation [8], [9], in humans between maternal nutrition during pregnancy and DNA methylation patterns [10] and between exposure to famine during the peri-conceptional period and DNA methylation patterns 60 years later [11], [12]. However, despite the comprehensive literature linking variation in DNA methylation to phenotypic variation in cancer or in the context of rare imprinting disorders [13] there is comparatively little evidence to date that variation in DNA methylation, or other epigenetic markings, are clearly associated with common complex diseases or the programming of such [14], although evidence is beginning to emerge in this regard [15]–[21]. Studies in rodent models have shown abnormalities of insulin secretion and action, appetite regulation, obesity, non-alcoholic fatty liver disease, hypertension and cardiovascular parameters following a variety of dietary challenges during pregnancy [3], [22]–[24] and the involvement of epigenetic processes is commonly postulated, with emerging empirical evidence to support this [25]. These observations have not yet been widely extended to human populations.

In order to investigate the potential causal role of epigenetic mechanisms linking early life exposures with later phenotype, specifically focusing on the early life programming of obesity, we have undertaken a study measuring methylation patterns in cord blood DNA and interrogated their relationship with later body size and composition. A targeted approach has been adopted whereby genes have been selected for DNA methylation analysis based upon their identification through gene expression analysis of children with high or low BMI at age 11–13 years. We hypothesise that DNA methylation patterns established *in utero* influence gene regulation and subsequently body composition, and explore this in human subjects.

**Figure 1:**
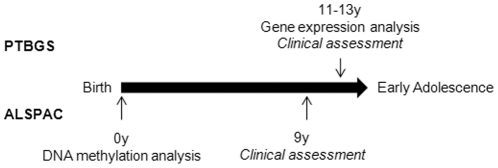
Overview of study design. Gene expression analysis was conducted on RNA samples collected at age 11–13 years when children in the Preterm Birth Growth Study (PTBGS) attended clinical assessment which included body composition measurement. Genes highlighted as being differentially expressed in relation to high/low BMI in this study group were then analysed in cord blood DNA samples from the Avon Longitudinal Study of Parents and Children (ALSPAC). Methylation levels were then analysed in relation to later body composition assessments carried out at 9 years in this study group.

The study design is summarised in Figure 1. Gene expression analysis of peripheral blood RNA from children age 11–13 years (from the Preterm Birth Growth Study) [26], grouped by body mass index (BMI), was conducted and used to identify genes which were up- or down-regulated. All genes showing evidence of differential expression and which were represented on the Illumina Cancer Panel I DNA methylation array were then interrogated to establish the relationship between DNA methylation status in cord blood and later body composition in a second cohort of 178 children from the Avon Longitudinal Study of Parents and Children (ALSPAC) [27].

The existence of inter-individual variation in DNA methylation makes it likely that many associations between DNA methylation and phenotype will be identified and reported, several of which will arise through chance and prove to be spurious. A series of statistical analyses was therefore applied, with increasing levels of stringency, to ensure that only robust associations were identified and to reduce the likelihood of highlighting false positive findings.

## Results

### Characteristics of the study populations

Characteristics of the PTBGS and ALSPAC subjects included in this study are shown in Table S1. The two contemporaneous study populations did not show differences in body mass index at age 11 years despite discordance in gestational age and birth weight although the PTBGS children had a higher fat mass when compared to the ALSPAC children at this age. (median [IQR]; PTBGS 14,051.2g [12,064.7], ALSPAC 10,403.8g [8,970.5], *p* = 0.021).

### Gene expression analysis

Fourteen samples were selected for gene expression analysis and summary details of the 2 groups (high/low BMI) are provided in Table S2. Three hundred and forty five genes were identified as being up-regulated and 190 genes down-regulated in children with high BMI. Of the 514 genes showing >1.2-fold differential expression (*p*<10^−4^), 29 (5.6% of differentially expressed genes) were represented on the Illumina GoldenGate Cancer Panel I array with a density of coverage of 1–3 CpG sites per gene. Of these 5 genes were dropped due to their methylation status being >99% or <1%, leaving 44 CpG sites in 24 genes that were taken forward for analysis in the ALSPAC cohort.

### Stringent statistical analysis of DNA methylation data

OLS regression analysis (I) resulted in many associations which were not replicable using data derived methods of standard errors, including robust regression techniques (II) and bootstrapping (III). Consequently, the number of associations reduced from analysis type I to analysis type III, although results were broadly similar between II and III. Only CpG sites which showed consistency across all analyses were considered to provide robust associations, therefore we present regression estimates from the bootstrapped analysis only (Table 1). A comparison of regression estimates from all three approaches is provided in Tables S3, S4, S5, S6.

**Table 1:** Bootstrap analysis of cord blood DNA methylation as a predictor of body composition (BMI, fat mass, lean mass and height) at age 9 years.

Gene	CpG site	BMI				Fat mass				Lean mass				Height			
		*adj age, sex, batch*				*adj age, sex, height, batch*				*adj age, sex, height, batch*				*adj age, sex, batch*			
		n	Est	SE	p	n	Est	SE	p	n	Est	SE	p	n	Est	SE	p
**Alkaline phosphatase**	ALPL_P	158	−0.14	0.22	0.52	150	−0.11	0.69	0.878	150	0.06	0.1	0.552	150	−0.15	0.04	**2E-04**
**Caspase 10**	CASP10_P2	75	−2.13	0.91	**0.02**	81	0.8	0.8	0.319	69	−0.38	0.34	0.261	69	−0.08	0.05	0.129
**Cyclin-dependent kinase inhibitor 1C**	CDKN1C_P2	157	2.08	0.97	**0.031**	149	5.16	2.48	**0.037**	149	0.86	0.39	**0.03**	149	−0.02	0.2	0.928
**Ephrin type-A receptor 1**	EPHA1_P	157	0.8	0.4	**0.048**	149	1.84	0.88	**0.036**	149	0.27	0.15	0.067	149	0.01	0.06	0.888
**HLA class II histocompatibility antigen DO beta chain**	HLA_DOB3	158	−0.31	0.24	0.187	150	−0.9	0.44	**0.039**	150	−0.06	0.11	0.618	150	0.02	0.03	0.624
**Interferon regulatory factor 5**	IRF5_P	156	0.75	1.05	0.471	148	2.55	2.51	0.31	148	0.6	0.47	0.204	148	−0.42	0.18	**0.022**
**Interferon regulatory factor 5**	IRF5_E	157	0.5	0.73	0.498	149	2.89	1.49	0.053	149	0.09	0.3	0.757	149	−0.29	0.13	**0.026**
**Matrix metalloproteinase 9**	MMP9_P	157	0.08	0.18	0.655	148	−0.12	0.57	0.836	148	0.17	0.08	**0.042**	148	−0.01	0.06	0.828
**Myeloproliferative leukemia virus oncogene**	MPL_P	158	0.1	0.11	0.353	150	0.15	0.33	0.642	150	0.11	0.05	**0.021**	150	−0.01	0.03	0.797
**Nidogen-2**	NID1_P	158	−0.48	0.3	0.101	150	−1.19	0.56	**0.035**	150	−0.1	0.14	0.827	150	0	0.04	0.984

The estimate provides the magnitude and direction of effect (%) on phenotype for a 1% increase in DNA methylation at that CpG site.

### DNA methylation at birth is associated with body size in childhood

We investigated the association between methylation at birth and BMI and its components at age 9 years, including measurements of lean mass, fat mass and height. Studying the 44 pre-selected probes corresponding to 24 candidate genes (Tables S3, S4, S5, S6), we observed a consistent association between methylation at *CDKN1C* and *EPHA1* CpG sites with BMI as well as fat mass at age 9 years (Table 1). This association was manifest as an estimated increase of 2.08% and 0.80% increase in BMI per 1% increase in methylation at *CDKN1C* and *EPHA1* respectively and an increase of 5.16% and 1.84% in fat mass per 1% increase in methylation respectively. Methylation at *CDKN1C* was also associated with lean mass, with an estimated 0.86% increase in lean mass per 1% increase in methylation. However, there was no evidence for association at either of these loci with height. Additional associations were observed between methylation at *CASP10* and BMI, methylation at *HLA-DOB* and *NID2* and fat mass and methylation at *MMP9* and *MPL* and lean mass. These and all other BMI, lean and fat mass specific findings however do not withstand multiple testing, and a larger sample is needed to confirm the robustness of the results. Adjustment of lean mass for fat mass was not reported given the high degree of colinearity of these variables due to the derivation of lean mass from fat mass measured by DXA.

**Figure 2:**
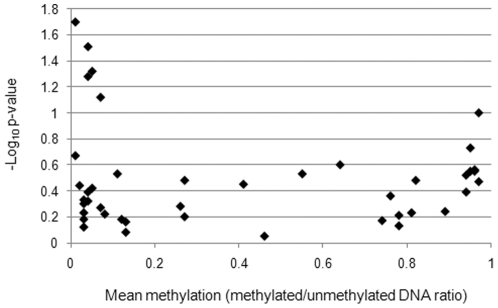
Distribution of methylation-phenotype associations. Distribution of −log_10_ *p*-values for bootstrap analysis of BMI and DNA methylation in cord blood DNA according to mean methylation levels at the 44 CpG sites analysed.

We found evidence for association of methylation at *ALPL* and *IRF5* with height (Table 1). For the *ALPL* locus, an increase in 1% methylation at birth was related to a 0.15% decrease in height at age 9 years for an average person at an average age (within the study cohort analysed). It is likely that the observed effects are specifically related to height, as the effects were still present when the model was fully adjusted for age, sex, batch, fat mass and lean mass (adjusted estimate mean (SD) = −0.07% (0.03), *p* = 0.032). Two CpG sites at the *IRF5* locus showed association with height; a CpG site in the promoter region and one in the first exon which were associated with a 0.42% and 0.29% decrease in height per 1% increase in methylation respectively (Table 1). Methylation at the two sites in this gene was correlated (rho = 0.442, *p*<0.001). Conservatively assuming up to 88 independent tests for body composition (mass and height independent analyses of 44 probes), only the identified association with methylation of *ALPL* and height withstands a correction for multiple testing (p_Corrected_ = 0.017). A plot of mean methylation versus −log_10_ *p*-value is shown in Figure 2, indicating a relatively even distribution of statistically significant observations in genes across high, intermediate and low levels of methylation (Figure 2).

The associations between increased DNA methylation, gene expression and indices of body composition for each of the 9 genes (10 CpG sites) showing some evidence of association between cord blood DNA methylation and later phenotype are summarised in Table 2.

**Table 2:** A summary of the relationship between increased DNA methylation in the genes listed (at a single CpG site defined by an Illumina GoldenGate probe), gene expression and indices of body composition.

Gene	Symbol	Direction of expression change in high BMI children	Phenotype influenced	Function¶
**Alkaline phosphatase**	*ALPL*	↓	Height	Bone mineralization
**Caspase 10**	*CASP10*	←–	BMI	Apoptosis
**Cyclin-dependent kinase inhibitor 1C**	*CDKN1C*	↓	BMI, Fat mass, Lean mass	Negative regulator of cell proliferation
**Ephrin type-A receptor 1**	*EPHA1*	↓	BMI, Fat mass	Development (nervous system)
**HLA class II histocompatibility antigen DO beta chain**	*HLADOB3*	↓	Fat mass	Antigen presentation
**Interferon regulatory factor 5**	*IRF5*	←–	Height	Cell growth, differentiation, apoptosis
**Matrix metalloproteinase 9**	*MMP9*	↓	Lean mass	Breakdown of extracellular matrix in tissue remodelling
**Myeloproliferative leukemia virus oncogene**	*MPL*	↓	Lean mass	Proliferation (bone marrow haemopoietic cells)
**Nidogen-2**	*NID1*	–	Fat mass	Cell interactions with extracellular matrix, adipogenesis

Brief details of the known gene function and evidence of literature pertinent to body composition and/or DNA methylation for each gene are summarised.

¶ As defined by GeneCards (http://www.genecards.org/).

## Discussion

An association between DNA methylation status at birth and body size in childhood was observed in 9 of the 24 (37.5%) genes selected *a priori* due to evidence of their differential gene expression in children with high BMI. This observation suggests that variation in DNA methylation patterns at birth in multiple target genes may influence body size in childhood. This association holds particular relevance for the role of epigenetic factors as mediators in early life programming of disease in later life. Genes that demonstrate perturbed expression in children with high BMI show signs of aberrant regulation at birth, albeit in two separate study populations. A recent study reported an association between loci displaying a high level of inter-individual variation in DNA methylation and BMI in adults [17], however this study was of elderly individuals so little can be inferred with respect to early life programming. More recent observations report an association between DNA methylation at a CpG site in the *RXR* gene promoter in DNA extracted from umbilical cord and later adiposity in children (aged 9 years) [18]. The findings of the current study are in accordance with this and offer additional support for a potential functional link between DNA methylation at birth and later adiposity, through interrogation of genes with perturbed gene expression profiles in children with high BMI.

The most robust observation in this study links DNA methylation at birth in the *ALPL* gene with decreased height at age 9 and this association is corroborated by clear evidence for a biological role of alkaline phosphatase (ALPL) in bone mineralization [28]. Furthermore, common polymorphisms in this gene have been associated with reduced bone mineral density, bone strength and skeletal size [29], [30]. Thus increased methylation of this gene could plausibly cause gene silencing leading to reduced gene function manifest as reduced frame size. To our knowledge this is the first evidence linking epigenetic variation to the determination of height. Recent and extensive investigations into the genetic determinants of height show clearly that this trait is the product of many loci acting in concert [31]. Further work exploring potential epigenetic perturbation of genes known to influence height through their polymorphic variation, which now number in the hundreds, may explain a further proportion of inter-individual variation in this trait.

Previous gene expression analysis studies of adipose tissue demonstrate marked differences in gene expression in obese subjects when compared to individuals with normal BMI [32], [33]. These observations in isolation do not allow one to dissect whether transcriptional changes are a cause or a consequence of the obese state. The data presented in this study extend these observations by demonstrating that DNA methylation differences are evident at birth in children who later develop high BMI and greater fat and lean mass in genes that are also differentially expressed in pre-adolescent children with high BMI. This supports the hypothesis that, at least in some biological pathways DNA methylation and gene expression changes *might* precede altered body composition and therefore obesity. Alternatively, the observed DNA methylation changes may be non-causal biomarkers un-related to gene expression and further studies are required to delineate this.

No association was observed between methylation status at the loci studied and birth weight (data not shown). This observation is in concordance with the observations of Tobi *et al.* who report no association of *in utero* growth restriction and methylation at birth at 4 loci perturbed by prenatal famine [34]. A recent study of 12 fetal cord blood samples however reports the converse, showing a correlation between both gene-specific and global DNA methylation (LINE-1) and infant birth weight [35]. Birth weight only correlates weakly with BMI at age 9 years (r = 0.127) in the whole ALSPAC cohort of >10,000 children and the sub-group used in this study is representative of this. Our observations suggest that the DNA methylation levels of genes interrogated does not impact significantly on growth *in utero* but that any effect on phenotype would appear to be acting during postnatal and childhood development.

A major component of adipogenesis is proliferation and differentiation of adipocytes. In the current study genes associated with cell cycle and proliferation (*CDKN1C*) showed evidence of differential methylation and expression. Previous studies have shown expression of *CDKN1C* to be up-regulated in omental adipose tissue of obese adults [36]. CDKN1C is a negative regulator of cell growth and proliferation and mutations are also implicated in the pathogenesis of Beckwith Wiedemann Syndrome, characterised by pre- and post-natal overgrowth [37]. Down-regulation of *CDKN1C*, as well as various other imprinted genes, has also been associated with a co-ordinated decline in postnatal growth rate [38]. The *NID2* gene, methylation of which was observed to be inversely associated with fat mass in this study, also plays a role in adipogenesis [39]. Ephrin type-A receptor 1 (EPHA1) has been implicated in the development of the nervous system but is also involved in the control of insulin signalling, which in turn plays a large role in body composition [40].

The matrix metalloproteinase family, which includes MMP9, are known to degrade the extracellular matrix [39] and increased levels of plasma MMP9 have been reported in both obese adults [41] and obese children [42]. Furthermore, Feinberg *et al.* [17] also identified *MMP9* as a locus displaying a consistent association between DNA methylation levels and BMI at two time points in older adults. In the current study no association was observed between *MMP9* methylation and BMI or fat mass but a negative correlation with height in childhood was observed.

With respect to the other genes showing evidence of an association between DNA methylation level, body size and composition, the biological evidence is less clear. To our knowledge, there are no known functional connections between *CASP10*, HLA-*DOB* or *MPL* and the determination of body size or composition.

Tissue specificity and the informativeness of non-target tissues such as peripheral blood leukocyte (PBL) DNA of methylation patterns is an area of much debate, although the use of PBL DNA [17], [43]–[48] or cord blood DNA [35], [49], [50], is commonplace in epidemiological studies as often this is the only source of DNA available. We observe some overlap in the genes demonstrating differential expression and methylation in cord blood DNA with previous published studies of adipose tissue. If considered at a pathway level we observe considerable overlap in the functional gene groups detected in cord blood DNA compared to adipose tissue (e.g. cell proliferation, apoptosis, adipogenesis). The present study underscores the utility of PBL DNA in defining biomarkers of methylation status that can be applied to epidemiological investigations to further harness information on the determinants and consequences of epigenetic variation and its impact on common complex diseases.

One further caveat of using DNA extracted from PBL or cord blood DNA is the relative contributions made by respective cell types, the ratio of which may vary for example in response to inflammation. Epigenetic signatures differ between cell types [51], although the overall impact of this is not considered to be substantial [45], [48]. The ‘synthesised’ longitudinal design adopted in the current study largely overcomes this caveat, at least in relation to establishing the relationship between methylation and phenotype. If for example inflammation, a common co-morbidity of obesity, were to impact upon blood cell type ratio and thereby distort methylation measurements this would be problematic in a cross-sectional study design. A recent study linking methylation changes in PBL DNA to obesity in adolescents in a cross sectional study reports exactly this; that one cannot infer causation from the observed association between methylation and the obesity phenotype [52]. Methylation analysis conducted at birth many years before the development of the phenotype overcomes this problem of an inability to exclude reverse causation. A further potential limitation is that the cord blood DNA samples used in this study were not selected at random but on the presence/absence of a particular prenatal exposure and postnatal phenotype. These variables were however not correlated with BMI and the distribution of the variables included in the current study was representative of the ALSPAC cohort.

Data analysis was initially based upon OLS linear regression, which has limitations when applied to DNA methylation data. The skewed distribution of methylation data results in heteroskedastic residuals which violate the assumptions required for hypothesis testing, the high degree of co-linearity between the exposures of interest and large leverage exerted by outliers due to the small sample size require that data interpretation is approached with caution. To this end we undertook stringent statistical tests including robust regression and bootstrapping. The number of associations falling below the standard statistical threshold of *p*<0.05 diminished as the level of stringency increased (Tables S3, S4, S5, S6). As both robust and bootstrapping methods provided overall consistent results, our findings suggest that robust techniques might be a valuable tool for high-throughput screening which can then be followed up by more computationally intensive and time-consuming bootstrapping.

A key question remaining is what factors determine the observed inter-individual variation in DNA methylation observed in cord blood DNA? A recent study of methylation patterns in DNA from twins from multiple tissue sources, including cord blood DNA, highlighted a large degree of variation in methylation even between MZ twins [50]. This suggests that the maternal *in utero* environment, including placentation and nutrient supply, may have an important influence on the neonatal epigenome. In a study of DNA methylation in maternal infant pairs Kile *et al* [49] found variable levels of correlation between methylation patterns of mothers and their offspring, indicative of some level of environmental contribution to this discordance. Many prenatal exposures have been linked to variation in DNA methylation including smoking, depression [53] and under- or over-nutrition [10], [11], [54], [55]. Folate exposure *in utero* has been implicated in the determination of spinal bone mineral density at age 9 years in the ALSPAC cohort [56], [57] which could in turn plausibly impact upon attained height. However, an assessment of the relationship between maternal folate intake during pregnancy, *MTHFR* genotype and body fat at age 9 years in this cohort showing no association between these factors indicates that folate exposure *in utero* is unlikely to explain the associations observed between cord blood methylation and fat mass in the current study [58].

A further and extremely relevant potential contributor to inter-individual variation in methylation at birth is the genetic determination of DNA methylation patterns. Variation in gene expression arising from allele-specific DNA methylation is well documented [59]–[61]. *Cis* (locally) acting genetic variation might determine DNA methylation levels and explain some of the inter-individual variation in methylation levels at birth. These changes would be expected to be stable over time and could not strictly be considered as ‘programmed’ events, rather inherited phenomena. The search for determinants of methylation variation at birth should include both genetic and environmental factors at play during the *in utero* period.

The current study has a number of limitations; the Illumina Cancer Panel I array was used to derive quantitative measures of gene-specific DNA methylation. This array only contained a fraction (29/514, 5.6%) of those genes observed to be differentially expressed in our high *vs* low BMI analysis. Furthermore, it is biased heavily towards tumour suppressor genes, oncogenes, DNA repair genes, cell cycle control, apoptosis and differentiation genes, X-linked and imprinted genes. This bias was overcome to a degree by the targeted approach and only using data from those genes implicated in the determination of body composition. Due to the technology employed, the reported associations rely on a few ‘representative’ CpG sites for each gene interrogated. A more comprehensive analysis of the gene regions of interest is required to gain a detailed understanding of the relationship between DNA methylation, gene regulation and phenotype.

As with all studies of this nature, multiple testing limits the robustness of the inferences that can be made about the observed associations. By applying robust statistical approaches we consider that we have minimised the potential for false positives, however further studies are required to establish the true validity of our observations. In addition to replication of the reported observations in other cohorts, further investigation of temporal variation in DNA methylation patterns from birth across childhood would be highly informative, together with examination of their relationship with developmental trajectories of body composition traits. The use of novel approaches for strengthening causal inference could also be usefully adopted [62]–[64]. Future studies should also include the analysis of DNA methylation using other methodological approaches to quantify DNA methylation, in particular the fine mapping of DNA methylation and DNA sequence variation across the genes identified in this study.

## Materials and Methods

### Study populations

Ethical approval was obtained from the ALSPAC Law and Ethics Committee and Local Research Ethics Committees in accordance with the guidelines of The Declaration of Helsinki. Written informed consent was obtained for all participants in the study.

#### Preterm Birth Growth Study (PTBGS)

Healthy preterm infants (≤34 weeks gestation) were recruited from the Special Care Baby Unit, Royal Victoria Infirmary, Newcastle upon Tyne, UK [26], and were followed up intensively through childhood. Clinical assessment including anthropometric and biochemical markers was undertaken at 11–13 years of age when blood samples were taken for DNA and RNA analysis. Of the original study cohort 24/83 individuals contributed to this study.

#### Avon Longitudinal Study of Parents and Children (ALSPAC)

Pregnant women from the Avon area in the South West of England whose expected dates of delivery were between April 1991 and December 1992 were invited to take part in the study, which was successful in recruiting over 14,000 pregnancies in this time period. ALSPAC is a prospective study and the extensive data collected during pregnancy and throughout childhood is described in detail elsewhere (http://www.alspac.bristol.ac.uk) [27]. Data pertaining to body composition at 9 years of age and relevant covariates were provided for use in this study. DNA extracted from cord blood was used for DNA methylation analysis (n = 178). Samples were selected as part of a prior study according to use (or not) of paracetemol during pregnancy and the presence/absence of asthma at age 91 months of age.

Summary details of the two study populations are provided in Table S1.

### RNA and DNA isolation

#### PTBGS

2.5 ml of blood were drawn into a PAXgene™ Blood RNA tube (PreAnalytiX QIAGEN GmbH), incubated at room temperature for 2 hours and then stored at −70°C until extracted. Total RNA was extracted from whole blood using the PAXgene™ Blood RNA System Kit following the manufacturer's instructions. RNA Integrity Number (RIN) was assessed using RNA Nano 6000 chips run on an Agilent 2100 Bioanalyzer (Agilent Technologies, Inc., Palo Alto, California, USA) and concentration determined using a NanoDrop™ ND-1000 spectrophotometer (NanoDrop Technologies, Thermo Fisher Scientific Inc., Waltham, MA, USA). Blood drawn into EDTA was used for DNA extraction using QIAamp DNA blood midi kit (Qiagen, Crawley, UK) following the manufacturer's protocol.

#### ALSPAC

DNA extraction applied standard phenol-chloroform extraction methods. DNA was re-suspended in 2 mM Tris and stored at −80°C.

### Gene expression analysis

Individuals were selected from the PTBGS who participated in a follow-up clinical examination of cardiometabolic traits during 2007–2008. Of the 24 subjects analysed, subjects passing QC representing the highest (n = 7) and lowest (n = 7) tertiles of distribution of BMI were compared; mean difference [SD] in BMI was 9.86 [2.37] kg/m^2^. Summary details of the two groups are provided in Table S2. No differences in height were evident between the two groups but they were, as expected, significantly discordant in weight, BMI and fat mass (*p*<0.002). RNA samples were sent to ServiceXS (Leiden, The Netherlands) for globin reduction, labelling, hybridization to Human NuGO-Hs1a520180 GeneChip arrays (covering 23,941 probes) and scanning of the arrays. Globin reduction was performed using GeneChip® Globin-Reduction kit (PreAnalytiX QIAGEN GmbH, Affymetrix Inc., Santa Clara, California) according to the manufacturer's instructions (Mat. No. 1029528) using Peptide Nucleic Acid (PNA) oligonucleotides complementary to human globin mRNA transcripts (PNA; GR PNA-L G2001 Panagene Inc., Korea) and Globin-Reduction RNA controls (No. 900586, PreAnalytiX QIAGEN GmbH, Affymetrix Inc., Santa Clara, California). Human NuGO-Hs1a520180 GeneChip CEL files were normalised in BioConductor (http://genomebiology.com/2004/5/10/R80/) using the GCRMA package. Genes with differential expression between BMI groups were identified with the RankProd package (<0.05 with 100 permutations of the class labels) (http://bioinformatics.oxfordjournals.org/content/22/22/2825.full). Annotations were attached to probe sets from the nugohs1a520180.db library (http://www.bioconductor.org/help/biocviews/2.6/data/annotation/html/nugohs1a520180.db.html). Raw and normalised data from the experiment was deposited in GEO (http://www.ncbi.nlm.nih.gov/geo/) with accession number GSE22013.

### DNA methylation analysis

500 ng genomic DNA was treated with sodium bisulphite to convert unmethylated cytosine to uracil using the EZ-96 DNA Methylation Kit™ (Zymo Research, Cambridge Biosciences, UK) according to the manufacturer's recommendations. Site-specific CpG methylation was analysed using 5 µl (at 50 ng/µl) bisulphite treated DNA using the GoldenGate® Cancer Panel I Array (Illumina Inc, USA) and the GoldenGate® Assay Kit with UDG on the Sentrix Universal-96 Array matrix v7A. This panel covers 1505 CpG sites selected from 807 genes, with a minimum of 719 genes overlapping with the expression array. The analysis was performed with background normalisation across two sample plates (batches) comprised of 96 samples each. The assay failed for 4 of the 1505 CpG sites. Arrays were imaged using a BeadArray scanner and image processing and intensity data extracted using Illumina BeadStudio v3.2, methylation module v3.2.5 custom software. The level of methylation at a given CpG site was determined by comparing the proportion of methylated to unmethylated signal (expressed as a beta score, 0–1). Four samples were assayed in duplicate in plate 1.

### Indices of body composition

#### PTBGS

Children were measured wearing a light hospital gown. Weight (to the nearest 0.1 kg) and height (to the nearest 0.1 cm) were measured using standard procedures, calibrated weighing scales (Tanita, Arlington Heights, Illinois) and a Harpenden stadiometer (Holtain Crosswell, Dyfed), and BMI (kg/m^2^) calculated. Fat and lean mass were assessed using total body scan mode on a GE Lunar iDXA machine and software version v11.

#### ALSPAC

Height was measured to the nearest 0.1 cm using a Harpenden stadiometer (Holtain Crosswell, Dyfed) and weight while wearing underwear was measured to the nearest 50 g using Tanita body fat analyser (model TBF 305, Tanita, Arlington Heights, Illinois). Fat mass and lean mass were assessed by whole body dual energy X-ray absorptiometry (DXA) (Prodigy scanner, Lunar Radiation Corp, Madison, Wisconsin, US).

### Data analysis

DNA methylation status in 178 cord blood DNA samples from the ALSPAC cohort was analysed for a total of 54 CpG sites in 29 genes. Where fewer than n = 178 were included in the analyses, this was due to missing phenotype data or poor call rates for particular probes. Distribution of DNA methylation at each CpG site was defined and those sites that were either fully methylated (mean>0.99) or fully un-methylated (mean<0.01) or with >25% samples with methylation values = 0 were dropped from the data set (10 CpG sites dropped, leaving 24 genes and 44 CpG sites included in remaining analysis). Percentage DNA methylation levels at the remaining 44 CpG sites were analysed to assess the relationship between methylation level and BMI, fat mass (g), lean mass (g) and height (cm) at age 9 years. BMI, fat and lean mass were log-transformed (natural logarithm) as they were not normally distributed (in agreement with numerous other studies where these variables commonly show a positively skewed distribution [65]). We selected several complementary analysis methods with increasing levels of robustness towards violations of normality, outlier effects and heteroskedasticity given the moderate sample numbers available for analysis (n∼150) in the present study. Specifically, we performed (I) ordinary least squared (OLS) regression and (II) robust regression [66], followed by (III) a non-parametric bootstrapping of the OLS model (I). Adjustments were made for age at clinic attendance (in months) and sex in all models due to their potential confounding influence on DNA methylation and outcome measures (i.e. BMI, height, lean and fat mass). Sample batch (plate) was also included as a covariate in all models due to its potential to cause substantial variation and bias in the DNA methylation data. Finally, height was included as a covariate in models assessing lean mass and fat mass due to its strong influence on these outcome measures. Further adjustment for height, lean mass and fat mass were made depending on the outcome variable being considered. Data were analysed using the statistical software package STATA (version 10.0) (Stata Corp, College Station, TX) and the R software (version 2.12.1).

## Supporting Information

Table S1 **Descriptive statistics for the two study cohorts.** The Preterm Birth Growth Study; DNA samples and outcome measures collected at age 11–13 y were used for gene expression analysis and the Avon Longitudinal Study of Parents and Children DNA samples extracted from cord blood were used for DNA methylation analysis with outcome measures collected at age 9 years. Data collected in ALSPAC at age 11years are provided for comparative purposes. Medians (inter-quartile range) are presented. P-values for Mann Whitney U test comparing variables in the Preterm Birth Growth Study and the ALSPAC cohort at age 11 y are provided.
(DOC)

Table S2 **Comparison of low and high BMI groups selected from the Preterm Birth Growth Study for gene expression analysis.** Mean (standard deviation) values are presented with t-test for between group comparisons, unless otherwise stated. *Median (inter-quartile range) presented and Mann-Whitney U statistic for between group comparisons.
(DOC)

Table S3 **Increase in % BMI for 1% increase in methylation.** Adjusted for age, sex and inter-plate variation.
(DOC)

Table S4 **Increase in % fat mass for 1% increase in methylation.** Adjusted for age, sex, height and inter-plate variation.
(DOC)

Table S5 **Increase in % lean mass for 1% increase in methylation.** Adjusted for age, sex, height and inter-plate variation.
(DOC)

Table S6 **Increase in % height for 1% increase in methylation.** Adjusted for age, sex and inter-plate variation.
(DOC)
