# Supplementary material for: DNA Methylation Patterns in Cord Blood DNA and Body Size in Childhood
Source: PLoS One. 2012 Mar 14;7(3):e31821. doi: 10.1371/journal.pone.0031821 (PMC3303769; doi:10.1371/journal.pone.0031821)
Supplement: Table S1 — Descriptive statistics for the two study cohorts. The Preterm Birth Growth Study; DNA samples and outcome measures collected at age 11–13 y were used for gene expression analysis and the Avon Longitudinal Study of Parents and Children DNA samples extracted from cord blood were used for DNA methylation analysis with outcome measures collected at age 9 years. Data collected in ALSPAC at age 11years are provided for comparative purposes. Medians (inter-quartile range) are presented. P-values for Mann Whitney U test comparing variables in the Preterm Birth Growth Study and the ALSPAC cohort at age 11 y are provided. (DOC) [file pone.0031821.s001.doc]

| Variable | Preterm Birth Growth Study | ALSPAC | | *p*-value |
| --- | --- | --- | --- | --- |
|  | **11-13 years** | **9 years** | **11 years** |  |
| **n** | 24 | 178 | - | - |
| **Sex (% male)** | 50 | 59 | - | - |
| **Birth weight (g)** | 1,317.5 (362.5) | 3,400.0 (705.0) | - | <0.0001 |
| **Gestational age (weeks)** | 30.1 (2.4) | 40.0 (2.0) | - | <0.0001 |
| **Height (cm)** | 152.0 (15.2) | 138.3 (8.1) | 149.8 (9.7) | 0.855 |
| **Weight (kg)** | 42.5 (15.4) | 33.3 (9.6) | 41.7 (13.2) | 0.846 |
| **Body mass index (kg/m^2^)** | 18.0 (9.2) | 17.3 (3.3) | 18.8 (4.9) | 0.772 |
| **Age (months)** | 144.8 (23.2) | 117.0 (3.0) | 141.0 (3.0) | 0.102 |
| **Fat mass (g)** | 14,051.2 (12,064.7) | - | 10,403.8 (8,970.5) | 0.021 |
| **Lean mass (g)** | 28,024.1 (8,771.5) | - | 28,802.5 (5,461.9) | 0.212 |
