# Supplementary material for: DNA Methylation Patterns in Cord Blood DNA and Body Size in Childhood
Source: PLoS One. 2012 Mar 14;7(3):e31821. doi: 10.1371/journal.pone.0031821 (PMC3303769; doi:10.1371/journal.pone.0031821)
Supplement: Table S2 — Comparison of low and high BMI groups selected from the Preterm Birth Growth Study for gene expression analysis. Mean (standard deviation) values are presented with t-test for between group comparisons, unless otherwise stated. *Median (inter-quartile range) presented and Mann-Whitney U statistic for between group comparisons. (DOC) [file pone.0031821.s002.doc]

| **Variable** | **Low BMI** | **High BMI** | **P-value** |
| --- | --- | --- | --- |
| **Height (cm)** | 150.1 (10.0) | 150.51 (10.7) | 0.938 |
| **Weight (kg)** | 35.4 (5.9) | 58.9 (11.0) | <0.0001 |
| **Body mass index (kg/m^2^)*** | 16.0 (1.45) | 25.8 (2.7) | 0.002 |
| **Age (months)** | 144.77 (11.2) | 152.3 (13.7) | 0.282 |
| **Fat mass (g)** | 9,499.5 (3,686.1) | 24,865.9 (4,428.2) | <0.0001 |
| **Lean mass (g)** | 23,570.3 (4,401.8) | 30,703.6 (8,688.4) | 0.025 |
|  |  |  |  |
