# Supplementary material for: DNA Methylation Patterns in Cord Blood DNA and Body Size in Childhood
Source: PLoS One. 2012 Mar 14;7(3):e31821. doi: 10.1371/journal.pone.0031821 (PMC3303769; doi:10.1371/journal.pone.0031821)
Supplement: Table S3 — Increase in % BMI for 1% increase in methylation. Adjusted for age, sex and inter-plate variation. (DOC) [file pone.0031821.s003.doc]

| **CpG site** | **n** | **OLS linear regression** | | | **Robust regression** | | | **Bootstrapped** | | |
| --- | --- | --- | --- | --- | --- | --- | --- | --- | --- | --- |
|  |  | Est | SE | p | Est | SE | p | Est | SE | p |
| **ALOX12_E** | 126 | -0.28 | 0.18 | 0.132 | -0.28 | 0.14 | **0.044** | -0.27 | 0.15 | 0.075 |
| **ALOX12_P** | 158 | -0.06 | 0.15 | 0.678 | -0.06 | 0.13 | 0.652 | -0.05 | 0.13 | 0.684 |
| **ALPL_P** | 158 | -0.14 | 0.19 | 0.448 | -0.14 | 0.23 | 0.530 | -0.14 | 0.22 | 0.520 |
| **BCL2A1_P** | 158 | -0.14 | 0.11 | 0.197 | -0.14 | 0.17 | 0.404 | -0.13 | 0.15 | 0.406 |
| **CASP10_E** | 111 | -1.50 | 1.24 | 0.228 | -1.50 | 1.24 | 0.229 | -1.51 | 1.21 | 0.212 |
| **CASP10_P** | 84 | 0.13 | 0.27 | 0.644 | 0.13 | 0.36 | 0.729 | 0.11 | 0.35 | 0.759 |
| **CASP10_P2** | 75 | -2.03 | 0.89 | **0.025** | -2.03 | 0.89 | **0.026** | -2.13 | 0.91 | **0.020** |
| **CAV1_P** | 158 | 0.21 | 0.28 | 0.463 | 0.21 | 0.40 | 0.612 | 0.20 | 0.38 | 0.597 |
| **CAV1_P2** | 158 | -0.06 | 0.24 | 0.808 | -0.06 | 0.22 | 0.792 | -0.05 | 0.21 | 0.827 |
| **CCL3_E** | 158 | -0.01 | 0.09 | 0.891 | -0.01 | 0.08 | 0.885 | -0.01 | 0.08 | 0.890 |
| **CCL3_P** | 158 | -0.28 | 0.20 | 0.154 | -0.28 | 0.29 | 0.330 | -0.27 | 0.26 | 0.305 |
| **CD9_E** | 156 | 0.83 | 0.47 | 0.080 | 0.83 | 1.04 | 0.423 | 0.70 | 0.85 | 0.410 |
| **CD9_P** | 158 | 0.21 | 0.16 | 0.176 | 0.21 | 0.23 | 0.353 | 0.21 | 0.21 | 0.334 |
| **CDKN1C_P** | 158 | 0.62 | 0.29 | **0.037** | 0.62 | 0.62 | 0.324 | 0.51 | 0.48 | 0.292 |
| **CDKN1C_P2** | 157 | 2.18 | 0.78 | **0.006** | 2.18 | 0.95 | **0.023** | 2.08 | 0.97 | **0.031** |
| **DSC2_E** | 141 | -0.24 | 0.34 | 0.476 | -0.24 | 0.27 | 0.375 | -0.24 | 0.27 | 0.381 |
| **DSC2_P** | 158 | -0.34 | 0.15 | **0.023** | -0.34 | 0.33 | 0.306 | -0.28 | 0.26 | 0.281 |
| **EPHA1_P** | 157 | 0.92 | 0.27 | **0.001** | 0.92 | 0.54 | 0.094 | 0.80 | 0.40 | **0.048** |
| **EVI2A_E** | 158 | -0.04 | 0.09 | 0.663 | -0.04 | 0.09 | 0.670 | -0.04 | 0.09 | 0.682 |
| **HLA_DOB1** | 158 | -0.08 | 0.09 | 0.357 | -0.08 | 0.11 | 0.435 | -0.08 | 0.10 | 0.436 |
| **HLA_DOB2** | 158 | -0.40 | 0.18 | **0.026** | -0.40 | 0.39 | 0.305 | -0.36 | 0.33 | 0.273 |
| **HLA_DOB3** | 158 | -0.38 | 0.14 | **0.006** | -0.38 | 0.31 | 0.229 | -0.31 | 0.24 | 0.187 |
| **IRF5_E** | 157 | 0.53 | 0.47 | 0.260 | 0.53 | 0.83 | 0.521 | 0.50 | 0.73 | 0.498 |
| **IRF5_P** | 156 | 0.77 | 0.84 | 0.359 | 0.77 | 1.11 | 0.487 | 0.75 | 1.05 | 0.471 |
| **KRT1_P** | 158 | -0.05 | 0.09 | 0.628 | -0.05 | 0.09 | 0.629 | -0.05 | 0.09 | 0.612 |
| **LCN2_P** | 158 | 0.06 | 0.11 | 0.606 | 0.06 | 0.10 | 0.585 | 0.05 | 0.10 | 0.588 |
| **LCN2_P2** | 157 | 0.09 | 0.08 | 0.278 | 0.09 | 0.08 | 0.253 | 0.09 | 0.08 | 0.249 |
| **MLLT4_P** | 155 | 0.87 | 0.70 | 0.215 | 0.87 | 1.64 | 0.597 | 0.67 | 1.23 | 0.584 |
| **MMP9_E** | 158 | -0.04 | 0.08 | 0.663 | -0.04 | 0.10 | 0.713 | -0.03 | 0.09 | 0.733 |
| **MMP9_P** | 156 | 0.08 | 0.21 | 0.706 | 0.08 | 0.18 | 0.666 | 0.08 | 0.18 | 0.655 |
| **MMP9_P2** | 121 | -1.16 | 1.24 | 0.352 | -1.16 | 1.23 | 0.348 | -1.11 | 1.23 | 0.365 |
| **MPL_P** | 158 | 0.10 | 0.12 | 0.412 | 0.10 | 0.11 | 0.371 | 0.10 | 0.11 | 0.353 |
| **MPL_P2** | 158 | -0.09 | 0.08 | 0.299 | -0.09 | 0.09 | 0.308 | -0.09 | 0.08 | 0.293 |
| **NID1_P** | 158 | -0.55 | 0.16 | **0.001** | -0.55 | 0.35 | 0.115 | -0.48 | 0.30 | 0.101 |
| **NID1_P2** | 158 | -0.44 | 0.21 | **0.039** | -0.44 | 0.44 | 0.313 | -0.38 | 0.35 | 0.284 |
| **NKX3_1_P** | 148 | 0.33 | 0.29 | 0.248 | 0.33 | 0.47 | 0.479 | 0.31 | 0.43 | 0.474 |
| **NKX3_1_P2** | 158 | -0.55 | 0.24 | **0.021** | -0.55 | 0.66 | 0.400 | -0.44 | 0.45 | 0.335 |
| **PMP22_P** | 158 | -0.45 | 0.21 | **0.031** | -0.45 | 0.46 | 0.323 | -0.39 | 0.36 | 0.278 |
| **PMP22_P** | 158 | -0.12 | 0.10 | 0.252 | -0.12 | 0.20 | 0.561 | -0.09 | 0.17 | 0.569 |
| **S100A12** | 158 | -0.12 | 0.11 | 0.267 | -0.12 | 0.13 | 0.346 | -0.12 | 0.12 | 0.331 |
| **TAL1_E** | 152 | 0.17 | 0.18 | 0.338 | 0.17 | 0.27 | 0.523 | 0.16 | 0.25 | 0.531 |
| **TAL1_P** | 118 | -0.66 | 0.42 | 0.119 | -0.66 | 0.32 | **0.044** | -0.66 | 0.34 | 0.052 |
| **TAL1_P2** | 155 | 0.05 | 0.13 | 0.697 | 0.05 | 0.11 | 0.646 | 0.05 | 0.11 | 0.635 |
| **VIM_P** | 154 | 0.81 | 0.71 | 0.260 | 0.81 | 1.58 | 0.612 | 0.57 | 1.28 | 0.656 |
|  |  |  |  |  |  |  |  |  |  |  |
