# Supplementary material for: DNA Methylation Patterns in Cord Blood DNA and Body Size in Childhood
Source: PLoS One. 2012 Mar 14;7(3):e31821. doi: 10.1371/journal.pone.0031821 (PMC3303769; doi:10.1371/journal.pone.0031821)
Supplement: Table S4 — Increase in % fat mass for 1% increase in methylation. Adjusted for age, sex, height and inter-plate variation. (DOC) [file pone.0031821.s004.doc]

| **CpG site** | **n** | **OLS linear regression** | | | **Robust regression** | | | **Bootstrapped** | | |
| --- | --- | --- | --- | --- | --- | --- | --- | --- | --- | --- |
|  |  | Est | SE | p | Est | SE | p | Est | SE | p |
| **ALOX12_E** | 121 | -0.56 | 0.56 | 0.313 | -0.56 | 0.50 | 0.259 | -0.61 | 0.51 | 0.231 |
| **ALOX12_P** | 150 | 0.06 | 0.44 | 0.889 | 0.06 | 0.44 | 0.888 | 0.06 | 0.44 | 0.899 |
| **ALPL_P** | 150 | -0.10 | 0.58 | 0.859 | -0.10 | 0.71 | 0.884 | -0.11 | 0.69 | 0.878 |
| **BCL2A1_P** | 150 | -0.54 | 0.32 | 0.087 | -0.54 | 0.36 | 0.134 | -0.53 | 0.35 | 0.125 |
| **CASP10_E** | 105 | -2.29 | 3.92 | 0.560 | -2.29 | 3.93 | 0.560 | -2.30 | 3.76 | 0.540 |
| **CASP10_P** | 81 | 0.82 | 0.77 | 0.287 | 0.82 | 0.80 | 0.304 | 0.80 | 0.80 | 0.319 |
| **CASP10_P2** | 69 | -1.13 | 3.07 | 0.714 | -1.13 | 2.85 | 0.693 | -1.20 | 2.99 | 0.687 |
| **CAV1_P** | 150 | 1.14 | 0.82 | 0.168 | 1.14 | 0.96 | 0.235 | 1.12 | 0.91 | 0.218 |
| **CAV1_P2** | 150 | -0.44 | 0.72 | 0.543 | -0.44 | 0.66 | 0.506 | -0.42 | 0.65 | 0.521 |
| **CCL3_E** | 150 | -0.18 | 0.27 | 0.513 | -0.18 | 0.26 | 0.493 | -0.18 | 0.25 | 0.476 |
| **CCL3_P** | 150 | -1.13 | 0.58 | 0.053 | -1.13 | 0.61 | 0.064 | -1.12 | 0.58 | 0.055 |
| **CD9_E** | 148 | 2.10 | 1.39 | 0.132 | 2.10 | 2.07 | 0.312 | 1.85 | 1.86 | 0.321 |
| **CD9_P** | 150 | 0.67 | 0.47 | 0.152 | 0.67 | 0.56 | 0.227 | 0.65 | 0.54 | 0.229 |
| **CDKN1C_P** | 150 | 1.45 | 0.88 | 0.101 | 1.45 | 1.24 | 0.247 | 1.22 | 1.06 | 0.248 |
| **CDKN1C_P2** | 149 | 5.37 | 2.34 | **0.023** | 5.37 | 2.41 | **0.027** | 5.16 | 2.48 | **0.037** |
| **DSC2_E** | 133 | -0.58 | 1.05 | 0.586 | -0.58 | 0.91 | 0.528 | -0.62 | 0.90 | 0.491 |
| **DSC2_P** | 150 | -0.93 | 0.43 | **0.032** | -0.93 | 0.54 | 0.089 | -0.85 | 0.49 | 0.080 |
| **EPHA1_P** | 149 | 2.05 | 0.82 | **0.014** | 2.05 | 0.93 | **0.029** | 1.84 | 0.88 | **0.036** |
| **EVI2A_E** | 150 | -0.23 | 0.27 | 0.388 | -0.23 | 0.26 | 0.375 | -0.23 | 0.26 | 0.378 |
| **HLA_DOB1** | 150 | -0.37 | 0.26 | 0.165 | -0.37 | 0.26 | 0.164 | -0.36 | 0.26 | 0.155 |
| **HLA_DOB2** | 150 | -1.14 | 0.53 | **0.032** | -1.14 | 0.72 | 0.117 | -1.10 | 0.66 | 0.096 |
| **HLA_DOB3** | 150 | -0.97 | 0.40 | **0.017** | -0.97 | 0.47 | **0.041** | -0.90 | 0.44 | **0.039** |
| **IRF5_E** | 149 | 2.96 | 1.42 | **0.038** | 2.96 | 1.64 | 0.072 | 2.89 | 1.49 | 0.053 |
| **IRF5_P** | 148 | 2.61 | 2.54 | 0.306 | 2.61 | 2.63 | 0.323 | 2.55 | 2.51 | 0.310 |
| **KRT1_P** | 150 | -0.29 | 0.28 | 0.296 | -0.29 | 0.28 | 0.300 | -0.29 | 0.28 | 0.294 |
| **LCN2_P** | 150 | -0.12 | 0.33 | 0.727 | -0.12 | 0.32 | 0.716 | -0.12 | 0.31 | 0.704 |
| **LCN2_P2** | 149 | 0.08 | 0.25 | 0.752 | 0.08 | 0.25 | 0.749 | 0.07 | 0.25 | 0.760 |
| **MLLT4_P** | 147 | 1.81 | 2.08 | 0.385 | 1.81 | 3.50 | 0.606 | 1.39 | 2.80 | 0.620 |
| **MMP9_E** | 150 | -0.25 | 0.24 | 0.297 | -0.25 | 0.25 | 0.321 | -0.25 | 0.25 | 0.318 |
| **MMP9_P** | 148 | -0.11 | 0.63 | 0.859 | -0.11 | 0.56 | 0.843 | -0.12 | 0.57 | 0.836 |
| **MMP9_P2** | 114 | -2.53 | 3.79 | 0.506 | -2.53 | 3.63 | 0.488 | -2.48 | 3.60 | 0.491 |
| **MPL_P** | 150 | 0.16 | 0.35 | 0.648 | 0.16 | 0.33 | 0.629 | 0.15 | 0.33 | 0.642 |
| **MPL_P2** | 150 | -0.38 | 0.25 | 0.121 | -0.38 | 0.23 | 0.093 | -0.39 | 0.22 | 0.082 |
| **NID1_P** | 150 | -1.26 | 0.49 | **0.011** | -1.26 | 0.51 | **0.015** | -1.19 | 0.56 | **0.035** |
| **NID1_P2** | 150 | -1.38 | 0.62 | **0.028** | -1.38 | 0.77 | 0.075 | -1.30 | 0.68 | 0.055 |
| **NKX3_1_P** | 140 | 1.40 | 0.86 | 0.106 | 1.40 | 1.04 | 0.182 | 1.40 | 0.98 | 0.154 |
| **NKX3_1_P2** | 150 | -1.46 | 0.70 | **0.038** | -1.46 | 1.10 | 0.185 | -1.34 | 0.91 | 0.141 |
| **PMP22_P** | 150 | -1.31 | 0.61 | **0.035** | -1.31 | 0.81 | 0.110 | -1.23 | 0.71 | 0.083 |
| **PMP22_P** | 150 | -0.38 | 0.30 | 0.215 | -0.38 | 0.41 | 0.360 | -0.34 | 0.36 | 0.353 |
| **S100A12** | 150 | -0.46 | 0.33 | 0.163 | -0.46 | 0.34 | 0.177 | -0.45 | 0.33 | 0.167 |
| **TAL1_E** | 144 | 0.68 | 0.52 | 0.198 | 0.68 | 0.58 | 0.248 | 0.65 | 0.56 | 0.250 |
| **TAL1_P** | 111 | -2.08 | 1.30 | 0.113 | -2.08 | 1.09 | 0.058 | -2.04 | 1.11 | 0.066 |
| **TAL1_P2** | 147 | 0.01 | 0.39 | 0.978 | 0.01 | 0.34 | 0.974 | 0.02 | 0.34 | 0.945 |
| **VIM_P** | 146 | 2.46 | 2.13 | 0.250 | 2.46 | 3.19 | 0.441 | 1.96 | 2.82 | 0.487 |
|  |  |  |  |  |  |  |  |  |  |  |
