# Supplementary material for: DNA Methylation Patterns in Cord Blood DNA and Body Size in Childhood
Source: PLoS One. 2012 Mar 14;7(3):e31821. doi: 10.1371/journal.pone.0031821 (PMC3303769; doi:10.1371/journal.pone.0031821)
Supplement: Table S5 — Increase in % lean mass for 1% increase in methylation. Adjusted for age, sex, height and inter-plate variation. (DOC) [file pone.0031821.s005.doc]

| **CpG site** | **n** | **OLS linear regression** | | | **Robust regression** | | | **Bootstrapped** | | |
| --- | --- | --- | --- | --- | --- | --- | --- | --- | --- | --- |
|  |  | Est | SE | p | Est | SE | p | Est | SE | p |
| **ALOX12_E** | 121 | -0.07 | 0.08 | 0.376 | -0.07 | 0.08 | 0.336 | -0.07 | 0.08 | 0.392 |
| **ALOX12_P** | 150 | 0.00 | 0.07 | 0.993 | 0.00 | 0.06 | 0.993 | 0.01 | 0.06 | 0.927 |
| **ALPL_P** | 150 | 0.06 | 0.09 | 0.501 | 0.06 | 0.10 | 0.566 | 0.06 | 0.10 | 0.552 |
| **BCL2A1_P** | 150 | 0.01 | 0.05 | 0.836 | 0.01 | 0.08 | 0.896 | 0.01 | 0.07 | 0.848 |
| **CASP10_E** | 105 | 0.21 | 0.55 | 0.706 | 0.21 | 0.76 | 0.785 | 0.24 | 0.67 | 0.717 |
| **CASP10_P** | 81 | -0.11 | 0.12 | 0.373 | -0.11 | 0.17 | 0.531 | -0.10 | 0.16 | 0.521 |
| **CASP10_P2** | 69 | -0.40 | 0.37 | 0.282 | -0.40 | 0.31 | 0.206 | -0.38 | 0.34 | 0.261 |
| **CAV1_P** | 150 | -0.03 | 0.12 | 0.787 | -0.03 | 0.18 | 0.849 | -0.04 | 0.16 | 0.815 |
| **CAV1_P2** | 150 | 0.09 | 0.11 | 0.392 | 0.09 | 0.10 | 0.373 | 0.10 | 0.10 | 0.341 |
| **CCL3_E** | 150 | 0.05 | 0.04 | 0.213 | 0.05 | 0.04 | 0.235 | 0.05 | 0.04 | 0.211 |
| **CCL3_P** | 150 | 0.02 | 0.09 | 0.796 | 0.02 | 0.13 | 0.856 | 0.03 | 0.12 | 0.805 |
| **CD9_E** | 148 | 0.27 | 0.21 | 0.197 | 0.27 | 0.40 | 0.504 | 0.24 | 0.35 | 0.498 |
| **CD9_P** | 150 | 0.05 | 0.07 | 0.445 | 0.05 | 0.10 | 0.598 | 0.05 | 0.10 | 0.579 |
| **CDKN1C_P** | 150 | 0.24 | 0.13 | 0.067 | 0.24 | 0.19 | 0.192 | 0.22 | 0.16 | 0.169 |
| **CDKN1C_P2** | 149 | 0.85 | 0.35 | **0.016** | 0.85 | 0.39 | **0.029** | 0.86 | 0.39 | **0.030** |
| **DSC2_E** | 133 | 0.22 | 0.15 | 0.142 | 0.22 | 0.14 | 0.119 | 0.22 | 0.14 | 0.109 |
| **DSC2_P** | 150 | -0.05 | 0.07 | 0.460 | -0.05 | 0.14 | 0.721 | -0.02 | 0.12 | 0.856 |
| **EPHA1_P** | 149 | 0.30 | 0.12 | **0.017** | 0.30 | 0.16 | 0.057 | 0.27 | 0.15 | 0.067 |
| **EVI2A_E** | 150 | 0.03 | 0.04 | 0.480 | 0.03 | 0.04 | 0.515 | 0.03 | 0.04 | 0.515 |
| **HLA_DOB1** | 150 | 0.01 | 0.04 | 0.792 | 0.01 | 0.05 | 0.837 | 0.01 | 0.05 | 0.809 |
| **HLA_DOB2** | 150 | -0.06 | 0.08 | 0.471 | -0.06 | 0.16 | 0.719 | -0.03 | 0.15 | 0.836 |
| **HLA_DOB3** | 150 | -0.08 | 0.06 | 0.176 | -0.08 | 0.12 | 0.480 | -0.06 | 0.11 | 0.618 |
| **IRF5_E** | 149 | 0.11 | 0.22 | 0.627 | 0.11 | 0.34 | 0.760 | 0.09 | 0.30 | 0.757 |
| **IRF5_P** | 148 | 0.59 | 0.38 | 0.122 | 0.59 | 0.48 | 0.220 | 0.60 | 0.47 | 0.204 |
| **KRT1_P** | 150 | 0.03 | 0.04 | 0.543 | 0.03 | 0.05 | 0.617 | 0.03 | 0.05 | 0.595 |
| **LCN2_P** | 150 | 0.07 | 0.05 | 0.147 | 0.07 | 0.05 | 0.179 | 0.07 | 0.05 | 0.156 |
| **LCN2_P2** | 149 | 0.06 | 0.04 | 0.113 | 0.06 | 0.04 | 0.131 | 0.06 | 0.04 | 0.118 |
| **MLLT4_P** | 147 | 0.48 | 0.31 | 0.126 | 0.48 | 0.55 | 0.389 | 0.45 | 0.46 | 0.327 |
| **MMP9_E** | 150 | 0.03 | 0.04 | 0.392 | 0.03 | 0.05 | 0.507 | 0.03 | 0.04 | 0.469 |
| **MMP9_P** | 148 | 0.16 | 0.09 | 0.074 | 0.16 | 0.08 | 0.054 | 0.17 | 0.08 | **0.042** |
| **MMP9_P2** | 114 | 0.12 | 0.50 | 0.812 | 0.12 | 0.48 | 0.805 | 0.12 | 0.47 | 0.801 |
| **MPL_P** | 150 | 0.11 | 0.05 | **0.033** | 0.11 | 0.05 | **0.029** | 0.11 | 0.05 | **0.021** |
| **MPL_P2** | 150 | 0.02 | 0.04 | 0.561 | 0.02 | 0.04 | 0.603 | 0.02 | 0.04 | 0.574 |
| **NID1_P** | 150 | -0.14 | 0.07 | 0.059 | -0.14 | 0.12 | 0.254 | -0.10 | 0.14 | 0.468 |
| **NID1_P2** | 150 | -0.06 | 0.09 | 0.535 | -0.06 | 0.18 | 0.737 | -0.03 | 0.15 | 0.827 |
| **NKX3_1_P** | 140 | -0.04 | 0.13 | 0.762 | -0.04 | 0.21 | 0.851 | -0.05 | 0.19 | 0.798 |
| **NKX3_1_P2** | 150 | -0.08 | 0.11 | 0.449 | -0.08 | 0.27 | 0.766 | -0.03 | 0.20 | 0.868 |
| **PMP22_P** | 150 | -0.08 | 0.09 | 0.379 | -0.08 | 0.18 | 0.648 | -0.06 | 0.16 | 0.719 |
| **PMP22_P** | 150 | -0.01 | 0.05 | 0.776 | -0.01 | 0.08 | 0.864 | 0.00 | 0.07 | 0.960 |
| **S100A12** | 150 | 0.04 | 0.05 | 0.376 | 0.04 | 0.06 | 0.492 | 0.04 | 0.06 | 0.463 |
| **TAL1_E** | 144 | -0.02 | 0.08 | 0.780 | -0.02 | 0.12 | 0.856 | -0.02 | 0.11 | 0.825 |
| **TAL1_P** | 111 | -0.04 | 0.18 | 0.820 | -0.04 | 0.17 | 0.809 | -0.03 | 0.16 | 0.834 |
| **TAL1_P2** | 147 | -0.01 | 0.06 | 0.807 | -0.01 | 0.06 | 0.819 | -0.01 | 0.06 | 0.834 |
| **VIM_P** | 146 | 0.25 | 0.32 | 0.434 | 0.25 | 0.59 | 0.668 | 0.20 | 0.51 | 0.690 |
|  |  |  |  |  |  |  |  |  |  |  |
