# Supplementary material for: DNA Methylation Patterns in Cord Blood DNA and Body Size in Childhood
Source: PLoS One. 2012 Mar 14;7(3):e31821. doi: 10.1371/journal.pone.0031821 (PMC3303769; doi:10.1371/journal.pone.0031821)
Supplement: Table S6 — Increase in % height for 1% increase in methylation. Adjusted for age, sex and inter-plate variation. (DOC) [file pone.0031821.s006.doc]

| **CpG site** | **n** | **OLS linear regression** | | | **Robust regression** | | | **Bootstrapped** | | |
| --- | --- | --- | --- | --- | --- | --- | --- | --- | --- | --- |
|  |  | Est | SE | p | Est | SE | p | Est | SE | p |
| **ALOX12_E** | 121 | -0.04 | 0.05 | 0.360 | -0.04 | 0.08 | 0.567 | -0.03 | 0.06 | 0.618 |
| **ALOX12_P** | 150 | -0.04 | 0.04 | 0.320 | -0.04 | 0.04 | 0.418 | -0.03 | 0.04 | 0.415 |
| **ALPL_P** | 150 | -0.15 | 0.05 | **0.001** | -0.15 | 0.04 | **0.0003** | -0.15 | 0.04 | **0.0002** |
| **BCL2A1_P** | 150 | 0.02 | 0.03 | 0.498 | 0.02 | 0.02 | 0.393 | 0.02 | 0.02 | 0.365 |
| **CASP10_E** | 105 | -0.58 | 0.33 | 0.083 | -0.58 | 0.43 | 0.178 | -0.55 | 0.40 | 0.167 |
| **CASP10_P** | 81 | -0.07 | 0.06 | 0.217 | -0.07 | 0.05 | 0.191 | -0.08 | 0.05 | 0.129 |
| **CASP10_P2** | 69 | -0.36 | 0.26 | 0.168 | -0.36 | 0.23 | 0.123 | -0.37 | 0.24 | 0.126 |
| **CAV1_P** | 150 | -0.07 | 0.07 | 0.300 | -0.07 | 0.07 | 0.289 | -0.07 | 0.06 | 0.272 |
| **CAV1_P2** | 150 | -0.02 | 0.06 | 0.712 | -0.02 | 0.06 | 0.722 | -0.02 | 0.06 | 0.726 |
| **CCL3_E** | 150 | -0.01 | 0.02 | 0.654 | -0.01 | 0.02 | 0.658 | -0.01 | 0.02 | 0.668 |
| **CCL3_P** | 150 | 0.03 | 0.05 | 0.583 | 0.03 | 0.04 | 0.529 | 0.03 | 0.04 | 0.496 |
| **CD9_E** | 148 | -0.11 | 0.12 | 0.340 | -0.11 | 0.11 | 0.330 | -0.13 | 0.11 | 0.252 |
| **CD9_P** | 150 | -0.04 | 0.04 | 0.354 | -0.04 | 0.03 | 0.292 | -0.04 | 0.03 | 0.267 |
| **CDKN1C_P** | 150 | -0.02 | 0.07 | 0.828 | -0.02 | 0.07 | 0.812 | -0.02 | 0.07 | 0.726 |
| **CDKN1C_P2** | 149 | 0.00 | 0.20 | 0.997 | 0.00 | 0.20 | 0.997 | -0.02 | 0.20 | 0.928 |
| **DSC2_E** | 133 | -0.16 | 0.08 | 0.067 | -0.16 | 0.08 | **0.047** | -0.15 | 0.08 | 0.058 |
| **DSC2_P** | 150 | 0.01 | 0.04 | 0.723 | 0.01 | 0.03 | 0.702 | 0.02 | 0.03 | 0.594 |
| **EPHA1_P** | 149 | 0.02 | 0.07 | 0.793 | 0.02 | 0.05 | 0.736 | 0.01 | 0.06 | 0.888 |
| **EVI2A_E** | 150 | 0.02 | 0.02 | 0.468 | 0.02 | 0.02 | 0.426 | 0.02 | 0.02 | 0.426 |
| **HLA_DOB1** | 150 | 0.01 | 0.02 | 0.793 | 0.01 | 0.02 | 0.785 | 0.01 | 0.02 | 0.768 |
| **HLA_DOB2** | 150 | 0.02 | 0.04 | 0.712 | 0.02 | 0.04 | 0.681 | 0.02 | 0.04 | 0.621 |
| **HLA_DOB3** | 150 | 0.01 | 0.03 | 0.748 | 0.01 | 0.04 | 0.752 | 0.02 | 0.03 | 0.624 |
| **IRF5_E** | 149 | -0.28 | 0.12 | **0.016** | -0.28 | 0.14 | **0.040** | -0.29 | 0.13 | **0.026** |
| **IRF5_P** | 148 | -0.42 | 0.20 | **0.040** | -0.42 | 0.18 | **0.022** | -0.42 | 0.18 | **0.022** |
| **KRT1_P** | 150 | 0.01 | 0.02 | 0.709 | 0.01 | 0.02 | 0.704 | 0.01 | 0.02 | 0.719 |
| **LCN2_P** | 150 | 0.03 | 0.03 | 0.292 | 0.03 | 0.03 | 0.288 | 0.03 | 0.03 | 0.278 |
| **LCN2_P2** | 149 | 0.03 | 0.02 | 0.206 | 0.03 | 0.02 | 0.211 | 0.03 | 0.02 | 0.190 |
| **MLLT4_P** | 147 | -0.19 | 0.17 | 0.262 | -0.19 | 0.19 | 0.314 | -0.22 | 0.18 | 0.222 |
| **MMP9_E** | 150 | 0.01 | 0.02 | 0.546 | 0.01 | 0.02 | 0.542 | 0.01 | 0.02 | 0.542 |
| **MMP9_P** | 148 | -0.01 | 0.05 | 0.819 | -0.01 | 0.06 | 0.845 | -0.01 | 0.06 | 0.828 |
| **MMP9_P2** | 114 | -0.36 | 0.30 | 0.239 | -0.36 | 0.33 | 0.273 | -0.35 | 0.31 | 0.264 |
| **MPL_P** | 150 | -0.01 | 0.03 | 0.786 | -0.01 | 0.03 | 0.798 | -0.01 | 0.03 | 0.797 |
| **MPL_P2** | 150 | 0.00 | 0.02 | 0.835 | 0.00 | 0.02 | 0.835 | 0.00 | 0.02 | 0.826 |
| **NID1_P** | 150 | 0.00 | 0.04 | 0.992 | 0.00 | 0.04 | 0.992 | 0.00 | 0.04 | 0.984 |
| **NID1_P2** | 150 | 0.04 | 0.05 | 0.495 | 0.04 | 0.05 | 0.472 | 0.04 | 0.05 | 0.387 |
| **NKX3_1_P** | 140 | -0.08 | 0.07 | 0.224 | -0.08 | 0.06 | 0.182 | -0.09 | 0.06 | 0.152 |
| **NKX3_1_P2** | 150 | 0.02 | 0.06 | 0.780 | 0.02 | 0.06 | 0.775 | 0.03 | 0.05 | 0.574 |
| **PMP22_P** | 150 | 0.03 | 0.05 | 0.519 | 0.03 | 0.05 | 0.497 | 0.04 | 0.04 | 0.379 |
| **PMP22_P** | 150 | 0.02 | 0.03 | 0.541 | 0.02 | 0.02 | 0.510 | 0.02 | 0.02 | 0.472 |
| **S100A12** | 150 | -0.01 | 0.03 | 0.733 | -0.01 | 0.02 | 0.706 | -0.01 | 0.02 | 0.710 |
| **TAL1_E** | 144 | -0.02 | 0.04 | 0.569 | -0.02 | 0.04 | 0.520 | -0.03 | 0.04 | 0.485 |
| **TAL1_P** | 111 | -0.09 | 0.11 | 0.421 | -0.09 | 0.11 | 0.411 | -0.09 | 0.10 | 0.366 |
| **TAL1_P2** | 147 | 0.02 | 0.03 | 0.498 | 0.02 | 0.03 | 0.469 | 0.02 | 0.03 | 0.476 |
| **VIM_P** | 146 | -0.20 | 0.17 | 0.263 | -0.20 | 0.18 | 0.283 | -0.22 | 0.17 | 0.205 |
|  |  |  |  |  |  |  |  |  |  |  |
